# Persistent Knee Monoarthritis as a Lyme Disease Presentation: A Diagnostic Challenge – Case Report

**DOI:** 10.1055/s-0043-1771487

**Published:** 2024-04-22

**Authors:** Rita Alçada, Nuno Pina Gonçalves, Rita Torres, Maria Helena Lourenço, Bárbara Flor de Lima, Francisco Guerra Pinto

**Affiliations:** 1 Serviço de Ortopedia, Hospital de Cascais Dr. José de Almeida, Cascais, Portugal; 2 Hospital Dr. Nélio Mendonça, Funchal, Madeira, Portugal; 3 Centro Hospitalar Lisboa Ocidental, Lisboa, Portugal; 4 Hospital Prof. Doutor Fernando Fonseca, Amadora, Portugal; 5 Hospital Ortopédico de Sant'Ana, Parede, Portugal

**Keywords:** arthritis, septic, case report, gonalgia, Lyme disease, monoarthritis

## Abstract

There are several differential diagnoses for knee monoarthritis. We report a patient with recurrent episodes of knee effusion, in which the non-specific clinical condition implied several diagnostic hypotheses, therapeutic inaccuracies, and a delay in implementing adequate treatment.

For more than 2 years, the patient underwent different Orthopedics and Rheumatology visits. She received multiple therapies, including a knee arthroscopy with partial meniscectomy with transient improvement of the complaints but not a definitive diagnosis. After collecting synovial fluid samples and successively negative microbiological tests, we established the diagnosis of overlap of septic arthritis by atypical microorganisms isolated from synovial tissue ( *Pantoea* spp. and *Staphylococcus saprophyticus* ) and Lyme arthritis. Washing and surgical debridement followed by targeted antibiotic therapy resulted in a transient response due to persistent infection (stage 3).

This case demonstrates the need for a multidisciplinary approach to knee monoarthritis.

## Introduction

Knee monoarthritis has several causes, implying a complex differential diagnosis 1 2 ( Table 1 ). Despite the wide literature recognition, Lyme monoarthritis remains a diagnostic challenge requiring high clinical suspicion due to the lack of epidemiological context (tick bite) or positive cultural tests. 1 2 3 4 The authors present a case of monoarthritis secondary to Lyme disease with a late diagnosis.

**Table 1:** Illustrative chart of the differential diagnosis of knee monoarthritis 1 6

Infectious	Bacterial (gonococcal and non-gonococcal) Viral Spirochetes (Lyme, Syphilis) Fungal
**Microcrystalline**	Drop Pseudogout Hydroxyapatite/Basic calcium phosphate Calcium oxalate
**Traumatic**	Occult/stress fracture Meniscal and/or ligament lesion Foreign body
**Inflammatory/Autoimmune**	Reactive arthritis Psoriatic arthritis Ankylosing spondylitis Rheumatoid arthritis Lupus Sarcoidosis Behçet disease
**Metabolic**	Hyperparathyroidism Hypothyroidism Hemochromatosis Acromegaly Amyloidosis
**Tumoral**	Osteochondroma Osteoid osteoma Pigmented villonodular synovitis Malignancy (primary or metastatic tumor)
**Miscellaneous**	Arthrosis Coagulopathy Vasculitis Osteonecrosis

## Case Report

**Fig. 1:**
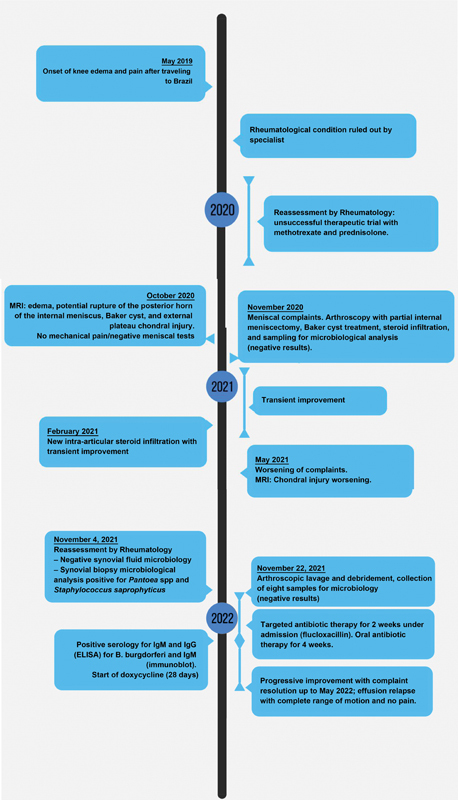
Timeline of the clinical course of the reported case. Description: Clinical evolution since the beginning of the condition. Complaints recurred in May 2022, with persistent effusion, functional impotence, and residual pain in August 2022. MRI, Magnetic resonance imaging.

A 39-year-old female with no relevant history presented a sudden onset of joint effusion and pain with a mixed rhythm in the right knee in May 2019. She denied complaints in other joints, fever, skin lesions, additional symptoms, tick bites, or exposure to other agents ( Fig. 1 ).

Given the predominantly inflammatory complaints, two rheumatologists evaluated the patient and performed a therapeutic trial with methotrexate and prednisolone for 4 months with no improvement.

**Fig. 2:**
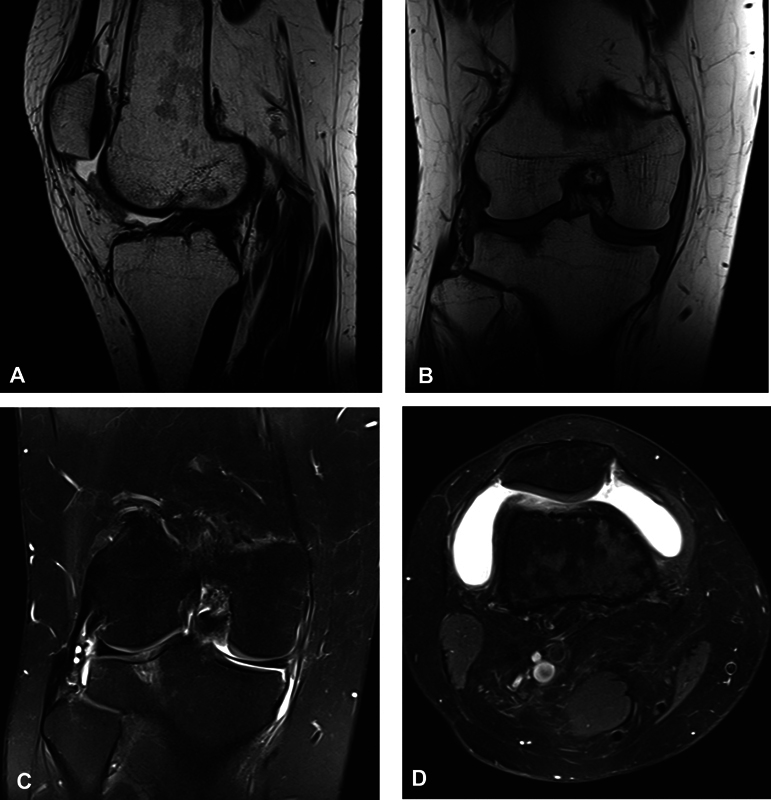
Magnetic resonance images. Description: A MRI from October 3, 2020, revealed intra-articular effusion, external tibial plateau injury, irregularity of the posterior horn of the internal meniscus, and a Baker cyst.A: T1-weighted sagittal section.B: T1-weighted coronal section.C: T2-weighted coronal section.D: T2-weighted axial section.

After 17 months, the patient underwent a magnetic resonance imaging (MRI) which confirmed joint effusion, a Baker cyst, a focus of medullary edema at the external plateau, and rupture of the internal meniscus ( Fig. 2 ). On this occasion, she had no meniscal-related complaints.

Eighteen months after symptom onset, persistent complaints and slightly worse mechanical issues led to a knee arthroscopy with synovial fluid sampling (negative bacteriological examination).

At the same time, the patient underwent microfractures of the external plateau injury, partial internal meniscectomy, excision of the valve system of the Baker cyst, and intra-articular infiltration with betamethasone. She presented a significant improvement in the postoperative period, with decreased edema and recovered range of motion.

Six months later, the patient had two recurrences of monoarthritis, interpreted as residual inflammation and controlled with new infiltrations. She underwent a new MRI revealing only the previously known chondral lesion. A third evaluation by Rheumatology was requested since the structural lesion did not justify the clinical condition.

In that assessment, 2.5 years after the condition onset, the patient underwent a laboratory follow-up to clarify the chronic knee monoarthritis of undetermined cause. Highlights included increased sedimentation rate (36 mm/h) and C-reactive protein (CRP) level, in addition to a negative autoimmunity panel and blood cultures. A new arthrocentesis and ultrasound-guided biopsy of the synovial membrane removed six membrane fragments for histopathological examination and five for bacteriological examination (including anaerobes, aerobes, mycobacteria, fungi, and parasites), and studies for *Neisseria gonorrhoeae* , *Chlamydia trachomatis* , and *Borrelia burgdorferi* . The synovial fluid was cloudy citrine yellow, and biochemistry results suggested chronic inflammatory arthropathy (22,240 cells/uL, 4.9 g/dL protein), with no casts and negative cultures. Histological examination of the synovial membrane suggested chronic synovitis with moderate inflammatory activity and predominantly lymphoid infiltrate, in addition to positive aerobic cultures for *Pantoea* spp. and oxacillin-sensitive *Staphylococcus saprophyticus* . The diagnosis of chronic septic arthritis with surgical indication was assumed. The patient underwent lavage and debridement, synovectomy, and arthroscopic sampling of tissue and synovial fluid for bacteriological examination (eight negative samples). She completed 2 weeks of intravenous flucloxacillin with a progressive resolution of effusion and pain, maintaining negative CRP during hospitalization. After discharge, the patient continued the oral antibiotic therapy for another 8 weeks after surgery (flucloxacillin, 2 g every 6 hours).

Six weeks after arthroscopic lavage, *B. burgdorferi* serology, performed in a reference laboratory, was positive for IgG and IgM by the ELISA method and confirmed by a positive IgM test by immunoblot. After multidisciplinary discussion (Rheumatology, Infectious Diseases, and Orthopedics), the antibiotic therapy switched to doxycycline for 28 days due to the diagnosis of Lyme disease. A new synovial biopsy was requested to detect *B. burgdorferi* DNA, which was negative (under antibiotic therapy).

The patient evolved favorably, with a progressive resolution of the condition. She resumed her work activity and walking without limitations. Six months after lavage, debridement, and initiation of antibiotic therapy, she presented a new episode of effusion with no pain or functional impotence. Repeated arthrocentesis revealed a negative *B. burgdorferi* DNA study, inconclusive serology for IgG and IgM by the ELISA method, and a confirmatory test by immunoblot showing positive IgM and negative IgG ( Table 2 ).

**Table 2:** Summary of microbiological tests

Date	Sampling method	Sample	Result
**November 22, 2020**	Arthroscopy	Synovial fluid	Negative
**November 03, 2021**	Ultrasound-guided arthrocentesis	Synovial fluid	Negative (aerobes, anaerobes, mycobacteria, parasites, fungi and screening for *N. gonorrhoeae* and *C. trachomatis* )
		Synovial biopsy	*Pantoea* spp. and *S. saprophyticus*
**22/11/2021**	Arthroscopy	Synovial fluid Synovial biopsy	Negative (total of 8 samples: anaerobes, aerobes, and mycobacteria)
**November-December 2021**	Serology	Serology	Positive IgM and IgG for *B. burgdorferi* (ELISA) and positive IgM (Immunoblot *)*
**January 13, 2022**	Ultrasound-guided arthrocentesis	Synovial fluid	Negative *B. burgdorferi* DNA screening (under antibiotic therapy)
**May 26, 2022**	Ultrasound-guided arthrocentesis	Synovial fluid	Negative *B. burgdorferi* DNA screening
	Serology	Serology	Inconclusive IgM and IgG results for *B. burgdorferi* (ELISA). Confirmatory test: positive IgM and negative IgG (Immunoblot *)*

Abbreviations *: C. trachomatis* , *Chlamydia trachomatis* ; *N. gonorrhoeae* , *Neisseria gonorrhoeae* ; *S. saprophyticus* , *Staphylococcus saprophyticus* .

## Discussion

The reported case illustrates the complexity of the differential diagnosis of knee monoarthritis due to Lyme disease. 1 2 Multiple negative samples and the lack of sensitive methods for *B. burgdoferi* identification led to the attribution of the patient's complaints to meniscal pathology, Baker cyst, cartilage damage, and postoperative residual inflammation. There was an overlap of septic arthritis and Lyme disease, and non-specific complaints implied a significant diagnostic delay. The identification of negative *S. coagulase* in deep biopsy is valuable, and contamination by *Pantoea* spp is a possibility. Only a multidisciplinary approach allowed adequate diagnosis and treatment more than 2 years after the symptom onset.

In Lyme disease, monoarthritis is frequently located in the knee and affects up to 60% of untreated patients; joint effusion is the most significant clinical finding. 5 6 7 Tick bites are often not documented. 5 6 The absence of erythema and heat, the analytical evaluation, and the biochemical and histological examinations findings also suggest Lyme arthritis. 1 5 7 8 It is noteworthy that, given the time, positivity for *B. burgdorferi* IgG was also expected in the confirmatory analysis (immunoblot). 6 The screening for *B. burgdorferi* DNA in synovial fluid after starting antibiotic therapy is usually negative. 5 6 In addition, culturing this pathogen is difficult, and culture sensitivity limits its use for diagnostic purposes. 1 5 Similarly to this case, the diagnosis usually relies on the seropositivity associated with arthritis. 5 Doxycycline is indicated in Lyme arthritis and results in excellent clinical response in most patients. 5 6 8

This persistent infection (stage 3) commonly affects the knee and causes frequent episodes of arthritis alternating with periods of remission. 7 8 9 This clinical course is relatively frequent, and it suggests that joint inflammation may persist despite the eradication of *B. burgdoferi* , leading to synovial damage and findings similar to those observed in other forms of chronic inflammatory arthritis. 7 9

Thus, in this case, the complex diagnostic strategy reiterates the importance of taking a multidisciplinary approach to patients with knee monoarthritis.

## References

[1] Maury E E Flores R H Acute monarthritis: diagnosis and management Prim Care 2006 33 03 779 793 17088160 10.1016/j.pop.2006.06.010

[2] Sack K Monarthritis: differential diagnosis Am J Med 1997 102(1A):30S 34S 9217557 10.1016/s0002-9343(97)00414-2

[3] Ma L Cranney A Holroyd-Leduc J M Acute monoarthritis: what is the cause of my patient's painful swollen joint? CMAJ 2009 180 01 59 65 19124791 10.1503/cmaj.080183 PMC2612045

[4] Siva C Velazquez C Mody A Brasington R Diagnosing acute monoarthritis in adults: a practical approach for the family physician Am Fam Physician 2003 68 01 83 90 12887114

[5] Arvikar S L Steere A C Diagnosis and treatment of Lyme arthritis Infect Dis Clin North Am 2015 29 02 269 280 25999223 10.1016/j.idc.2015.02.004 PMC4443866

[6] Matzkin E Suslavich K Curry E J Lyme Disease Presenting as a Spontaneous Knee Effusion J Am Acad Orthop Surg 2015 23 11 674 682 26416616 10.5435/JAAOS-D-14-00388

[7] Lochhead R B Strle K Arvikar S L Weis J J Steere A C Lyme arthritis: linking infection, inflammation and autoimmunity Nat Rev Rheumatol 2021 17 08 449 461 34226730 10.1038/s41584-021-00648-5 PMC9488587

[8] Miller J B Aucott J N Stages of Lyme Arthritis J Clin Rheumatol 2021 27 08 e540 e546 32815909 10.1097/RHU.0000000000001513

[9] Bennett J E Dolin R Blaser M J Mandell. Douglas, and Bennett's principles and practice of infectious diseases Philadelphia Elsevier 2020

