# Supplementary material for: Persistent Knee Monoarthritis as a Lyme Disease Presentation: A Diagnostic Challenge – Case Report
Source: Rev Bras Ortop (Sao Paulo). 2024 Apr 22;59(Suppl 2):e149–54. doi: 10.1055/s-0043-1771487 (PMC11679618; doi:10.1055/s-0043-1771487)
Supplement: Related texts

# Research article: Monoartrite persistente do joelho como apresentação de doença de Lyme: Um desafio diagnóstico – Relato de caso

**Keywords:** artrite séptica, doença de Lyme, gonalgia, monoartrite, relato de caso

## Abstract

Na investigação da monoartrite do joelho deverão ser considerados vários diagnósticos diferenciais. Relata-se o caso de uma doente com episódios recorrentes de derrame do joelho, em que a clínica inespecífica implicou várias hipóteses diagnósticas, imprecisões terapêuticas e um atraso na implementação de terapêutica adequada.

Durante mais de 2 anos, a doente foi avaliada em diferentes consultas de Ortopedia e Reumatologia. Realizou múltiplas terapêuticas incluindo uma artroscopia do joelho com meniscectomia parcial, com melhoria transitória das queixas, contudo sem diagnóstico definitivo. Após coleta de amostras de líquido sinovial com exames microbiológicos sucessivamente negativos, verificou-se uma sobreposição de artrite séptica por microrganismos atípicos isolados no tecido sinovial ( *Pantoea spp* . e *Staphylococcus saprophyticus* ) e artrite de Lyme. Foi realizada lavagem e desbridamento cirúrgico, seguido de antibioterapia dirigida com resposta transitória, por infeção persistente (estadio 3).

O caso apresentado demonstra a necessidade de uma abordagem multidisciplinar da monoartrite do joelho.

## Introdução

A monoartrite do joelho tem diversas causas, implicando um diagnóstico diferencial complexo 1 2 ( Table 1 ). A monoartrite de Lyme, apesar de amplamente reconhecida na literatura, continua a representar um desafio diagnóstico que requer alta suspeição clínica, por ser frequente a ausência de contexto epidemiológico (picada de carrapato) ou de exames culturais positivos. 1 2 3 4 Os autores apresentam um caso de monoartrite secundária a doença de Lyme com diagnóstico tardio.

**Table 1:** Quadro ilustrativo do diagnóstico diferencial de monoartrite do joelho 1 6

Infeciosa	Bacteriana (gonocócica e não-gonocócica) Viral Espiroquetas (Lyme, Sifilis) Fungíca
**Microcristalina**	Gota Pseudogota Hidroxiapatite/Fosfato de cálcio básico Oxalato de cálcio
**Traumática**	Fractura oculta / de stress Leão meniscal e/ou ligamentar Corpo estranho
**Inflamatória / Auto-Imune**	Artrite reactiva Artrite psoriática Espondilite anquilosante Artrite Reumatóide Lúpus Sarcoidose Behçet
**Metabólica**	Hiperparatiroidismo Hipotiroidismo Hemocromatose Acromegália Amiloidose
**Tumoral**	Osteocondroma Osteoide osteoma Sinovite vilonodular pigmentada Tumor maligno (primário ou metastático)
**Outros**	Artrose Coagulopatia Vasculite Osteonecrose

## Relato do Caso

Paciente do sexo feminino, de 39 anos, sem antecedentes relevantes, apresentou-se com quadro súbito de derrame articular e dor de ritmo misto no joelho direito, em maio de 2019. Negava queixas em outras articulações, febre, lesões cutâneas ou outra sintomatologia, bem como mordida por carrapato ou outro agente ( Fig. 1 ).

**Fig. 1:** *Timeline* da evolução do caso relatado. Descrição: Evolução clínica desde o início do quadro. Destaca-se recidiva das queixas em Maio de 2022, mantendo derrame com impotência funcional e dor residuais em Agosto de 2022.

Dadas as queixas predominantemente inflamatórias, foi avaliada por dois reumatologistas, tendo realizado prova terapêutica com metotrexato e prednisolona durante 4 meses, sem melhoria.

Após 17 meses realizou Ressonância Magnética (RMN) que confirmou derrame articular e a presença de cisto de *Baker* , foco de edema medular do planalto externo e rotura do menisco interno ( Fig. 2 ). Nesta ocasião sem queixas meniscais.

**Fig. 2:** Imagens de ressonância magnética . Descrição: RMN de 3 de Outubro de 2020. É possível observar derrame intra-articular, lesão do planalto tibial externo, irregularidade do corno posterior do menisco interno e cisto de Baker.A: Corte sagital, T1B: Corte coronal T1C: Corte coronal T2D: Corte axial T2

Após 18 meses do início do quadro, por persistência de queixas e discreto agravamento das queixas mecânicas foi submetida a artroscopia do joelho com coleta de líquido sinovial (exame bacteriológico negativo).

Concomitantemente, foram realizadas microfracturas da lesão do planalto externo, meniscectomia parcial interna, excisão de sistema valvular do cisto de Baker e infiltração intra-articular com betametasona. No período pós-operatório apresentou melhoria significativa, com diminuição do edema e recuperação do arco de mobilidade.

Após seis meses apresentou duas recidivas da monoartrite, interpretadas como inflamação residual e controladas com novas infiltrações. Repetiu RMN, observando-se apenas a já conhecida lesão condral. Não sendo justificável o quadro clínico por lesão estrutural, foi solicitada uma terceira avaliação pela Reumatologia.

Nessa observação, 2 anos e meio após início do quadro, realizou controle laboratorial para esclarecimento do quadro de monoartrite crônica do joelho de causa indeterminada. Destacavam-se: aumento da velocidade de sedimentação (36mm/h), e proteína C reactiva (PCR), estudo de doenças auto-imunes e hemoculturas negativos. Foi realizada nova artrocentese e biópsia da membrana sinovial ecoguiada, com remoção de 6 fragmentos de membrana para exame histopatológico e 5 para bacteriológico (de anaeróbios, aeróbios, micobacterias, fungos e parasitas), pesquisa de *Neisseria Gonorrhoeae, Chlamydia trachomatis e Borrelia Burgdorferi.* O líquido sinovial apresentava-se amarelo citrino turvo com bioquímica sugestiva de artropatia inflamatória crônica (22240cél/uL, 4.9g/dL de proteínas), sem cristais e com culturas negativas. O exame histológico da membrana sinovial foi sugestivo de sinovite crônica com moderada atividade inflamatória e infiltrado predominantemente linfoide, mas com cultura em aerobiose positiva para *Pantoea spp* . e *Staphylococcus saprophyticus* , sensíveis à oxacilina. Assumiu-se o diagnóstico de artrite séptica crônica com indicação cirúrgica. A paciente foi submetida a lavagem e desbridamento, sinovectomia e coleta de tecido e líquido sinoviais para exame bacteriológico (8 amostras negativas) por via artroscópica. Cumpriu 2 semanas de flucloxacilina endovenosa com resolução progressiva do derrame e dor, mantendo PCR negativa durante a internação hospitalar. Teve alta com indicação para manter antibioterapia oral durante mais 8 semanas pós cirurgia (flucloxacilina 2g de 6 em 6h).

Seis semanas após a lavagem artroscópica, obteve-se o resultado da sorologia de *B. burgdorferi* realizada em laboratório de referência, que foi positiva para IgG e IgM por método ELISA, com teste confirmatório IgM por *Immunoblot* positivo *.* Após discussão multidisciplinar (Reumatologia, Infeciologia e Ortopedia), a antibioterapia foi alterada para doxicicilina durante 28 dias, admitindo-se Doença de *Lyme.* Foi realizada nova biópsia da sinovial para pesquisa de DNA de *B. burgdorferi* , que foi negativa (sob antibioterapia).

A doente evoluiu favoravelmente, com resolução progressiva do quadro, retomou a atividade laboral e a deambulação sem limitações. Aos 6 meses pós lavagem, desbridamento e início de antibioterapia destaca-se novo episódio de derrame, sem dor ou impotência funcional. Repetiu artrocentese mantendo pesquisa de DNA de *B. burgdorferi* negativa, com serologia inconclusiva para IgG e IgM por método ELISA, com teste confirmatório por *Immunoblot* IgM positivo e IgG negativo ( Table 2 ).

**Table 2:** Quadro resumo com os exames microbiológicos realizados

Data	Método de Colheita	Amostra	Resultado
**22/11/2020**	Artroscopia	Líquido sinovial	Negativo
**03/11/2021**	Artrocentese ecoguiada	Líquido sinovial	Negativo (aeróbios, anaeróbios, micobactérias, parasitas, fungos e pesquisa de *N. gonorrhoeae e C. trachomatis* )
		Biópsia sinovial	*Pantoea spp.* e *S. Saprophyticus*
**22/11/2021**	Artroscopia	Líquido Sinovial Biópsia Sinovial	Negativo (total de 8 colheitas: anaeróbios, aeróbios e micobactérias)
**Novembro-Dezembro 2021**	Serologia	Serologia	IgM e IgG positivas para *B. burgdorferi* (ELISA) e IgM positiva ( *Immunblot)*
**13/01/2022**	Artrocentese ecoguiada	Líquido sinovial	Pesquisa de DNA de *B. burgdorferi* negativa (sob antibioterapia)
**26/05/2022**	Artrocentese ecoguiada	Líquido sinovial	Pesquisa de DNA de *B. burgdorferi* negativa
	Serologia	Serologia	IgM e IgG inconclusivos para *B. burgdorferi* (ELISA). Teste confirmatório com IgM positiva e IgG negativa ( *Immunblot)*

Legenda: C. trachomatis, Chlamydia trachomatis; N. gonorrhoeae, Neisseria gonorrhoeae; S. Saprophyticus, Staphylococcus Saprophyticus.

## Discussão

O caso relatado ilustra a complexidade do diagnóstico diferencial de monoartrite do joelho por Doença de Lyme. 1 2 As múltiplas colheitas negativas e a ausência de métodos sensíveis de identificação de *B. burgdoferi* levaram à atribuição das queixas da doente à patologia meniscal, cisto de Baker, lesão da cartilagem e inflamação residual pós-operatória. Verificou-se uma sobreposição de artrite séptica e doença de Lyme em que as queixas inespecíficas implicaram um atraso significativo no diagnóstico. É valorizável a identificação de S. coagulase negativo em biópsia profunda, sendo que é possível contaminação por *Pantoea spp* . Apenas uma abordagem multidisciplinar permitiu o diagnóstico e tratamento adequados mais de dois anos após o início das queixas.

A monoartrite na doença de Lyme localiza-se mais frequentemente no joelho e afeta até 60% dos doentes não tratados, sendo o derrame articular o principal achado clínico. 5 6 7 Frequentemente não é documentada a mordedura de carrapato. 5 6 A ausência de rubor e calor, a avaliação analítica e os exames bioquímico e histológico no caso descrito são também sugestivos de artrite de Lyme. 1 5 7 8 Destaca-se que, dado o tempo de evolução, seria de esperar positividade para IgG de *B. burgdorferi* também na análise confirmatória ( *Immunoblot* ). 6 A pesquisa de DNA de *B. burgdorferi* no líquido sinovial após início de antibioterapia é frequentemente negativa. 5 6 Também a cultura deste microrganismo é difícil e a sua sensibilidade limitam a sua utilização para diagnóstico. 1 5 Como neste caso, o diagnóstico é geralmente feito com base na seropositividade associada a artrite. 5 A doxiciclina está indicada na artrite de Lyme, com excelente resposta clínica na maioria dos doentes. 5 6 8

Estamos perante uma infeção persistente (estadio 3), sendo o joelho mais comumente afetado, com episódios frequentes de artrite alternados com períodos de remissão. 7 8 9 Este tipo de evolução é relativamente frequente e sugere que a inflamação articular pode manter-se apesar da irradicação da *Borrelia burgdoferi* , condicionando uma lesão da sinovial com achados semelhantes aos encontrados noutras formas de artrite inflamatória crônica. 7 9

Assim, a complexidade da marcha diagnóstica do caso apresentado reitera a importância de realizar uma abordagem multidisciplinar dos doentes com monoartrite do joelho.
